# Pre-treatment With Ranibizumab Aggravates PDT Injury and Alleviates Inflammatory Response in Choroid-Retinal Endothelial Cells

**DOI:** 10.3389/fcell.2020.00608

**Published:** 2020-07-09

**Authors:** Yang Liu, Min Zhu, Ruowen Gong, Xin Wang, Lei Li, Gezhi Xu

**Affiliations:** ^1^Shanghai Key Laboratory of Visual Impairment and Restoration, Eye & ENT Hospital, Fudan University, Shanghai, China; ^2^NHC Key Laboratory of Myopia (Fudan University), Key Laboratory of Myopia, Chinese Academy of Medical Sciences, Shanghai, China

**Keywords:** PDT, anti-VEGF, pre-treatment, necroptosis, NLRP3/IL-1β inflammatory response

## Abstract

Polypoidal choroidal vasculopathy (PCV) is the predominant subtype of exudative age-related macular degeneration in Asians. Although photodynamic therapy (PDT) is widely used for PCV treatment, its long-term beneficial effects are unsatisfactory. Accumulating clinical investigations suggest that combined therapy with anti-vascular endothelial growth factor (anti-VEGF) and PDT is superior to PDT monotherapy. However, the optimal time of anti-VEGF before or after PDT remains controversial, hence it needs to further explore the mechanism underlying combined therapy. PDT causes selective damage to endothelial cells, which determines its angio-occlusive efficiency, yet the impact of anti-VEGF on PDT-induced endothelial injury is unclear. Here, we found that pre- compared to post-treatment with anti-VEGF ranibizumab (rani) significantly aggravates PDT injury in the rhesus macaque choroid-retinal endothelial (RF/6A) cell line. PDT activates apoptosis, necroptosis and NLRP3 inflammasome in RF/6A cells. Pre-treatment with rani promotes PDT-caused apoptosis via triggering caspase 8-mediated extrinsic apoptosis, and caspase 8 might also play a pivotal role in the rani’s function of suppressing PDT-induced necroptosis and NLRP3 inflammasome activation. Our results implicate that pre-treatment with rani may enhance the angio-occlusive efficiency of PDT and alleviate endothelial inflammatory response, which gives it a great advantage over post-treatment.

## Introduction

Age-related macular degeneration (AMD) is a leading cause of visual loss across the world. However, AMD in Asians differs from that in Westerns in terms of epidemiology, pathogenesis, clinical presentation and treatment (Lim et al., 2013). Particularly, polypoidal choroidal vasculopathy (PCV) is the predominant subtype of exudative AMD in Asians, in contrast to choroidal neovascularization (CNV) secondary to AMD in Caucasian populations. Those two subtypes respond quite differently to treatments. The standard of care for CNV-AMD is anti-vascular endothelial growth factor (anti-VEGF) therapy, whereas the optimal treatment guidelines for PCV have not been well established. It is projected that Asia will contribute the highest global prevalence of AMD by 2040 (Wong et al., 2014), hence it is necessary and urgent to develop better treatment for PCV.

PCV, first identified and reported by Yannuzzi et al. (1990), is characterized by the presence of terminal dilatations of the abnormal branching choroidal vascular network referred to as polyps (Wong et al., 2016). The current treatment options for PCV include intravitreal anti-VEGF and verteporfin photodynamic therapy (PDT). PDT has been widely considered as the mainstay of treatment for PCV due to the regression of polyps, though it leads to increased levels of VEGF and inflammation, which both exacerbate the disease and accelerate PCV recurrence (Wong et al., 2016). To manage recurrence, PDT retreatment is needed, and repeated PDT may result in choroid or retina atrophy, which causes permanent visual impairment. As such, the long-term beneficial effects after PDT monotherapy are unsatisfactory.

Accumulating clinical investigations have suggested that combined therapy with anti-VEGF and PDT is superior to PDT monotherapy, as anti-VEGF neutralizes the extra VEGF produced after PDT and thereby reduces angiogenesis recurrence. There are now three anti-VEGF drugs in use: aflibercept (afli), ranibizumab (rani) and bevacizumab (beva). Rani and beva both are anti-VEGF antibodies and were developed several years before afli, a decoy VEGF receptor. Although anti-VEGF antibodies have a much longer history of application in combined therapy, the optimal time of antibodies before or after PDT is still controversial. Most of those studies utilized intravitreal antibodies before PDT (Gomi et al., 2010, 2015; Nowak-Sliwinska et al., 2010; Park and Kim, 2012; Sagong et al., 2012; Saito et al., 2012, 2014; Tomita et al., 2012; Jeon et al., 2013; Sato et al., 2013; Yoshida et al., 2013; Sakurai et al., 2014), and a few studies applied antibodies after PDT (Lai et al., 2011; Ho et al., 2014; Zhao et al., 2017) or in the same day (Kim et al., 2011; Moon et al., 2011; Ricci et al., 2012; Hata et al., 2015). Due to that there are great differences among them in regarding to anti-VEGF antibodies, time interval between PDT and intravitreal antibodies, PDT protocol and retreatment protocols, it is unable to conduct sub-group analysis and determine the best option. Furthermore, without a well-established PCV animal model, it is impossible to compare the therapeutic effects of various treatment protocols directly. Therefore, to optimize treatment for PCV, it needs to further explore the mechanism underlying combined treatment with PDT and anti-VEGF antibodies.

PDT with photosensitizers results in selective damage to endothelial cells in the abnormal blood vessels, which determines its angio-occlusive efficiency. VEGF has been considered as a crucial pro-survival factor, and blocking its function enhances the susceptibility of endothelial cells to injury (Domigan et al., 2015; Watson et al., 2016). However, the impacts of anti-VEGF antibodies on PDT-induced endothelial cell injury remain unclear. Previous studies have implied that PDT causes several types of cell death, including apoptosis, necrosis, and autophagy (Buytaert et al., 2007). Recently, a new form of cell death called necroptosis was discovered, which is a subset of necrosis and causes the release of cellular contents and induces inflammation in neighboring cells (Zhang et al., 2018). Nonetheless, unlike conventional necrosis is uncontrollable, necroptosis is a tightly regulated process: its initiation dependents on the kinase activities of receptor interacting protein kinase (RIP) 1 and RIP3, and its execution is mediated by phosphorylated MLKL (p-MLKL) (Silke et al., 2015). Accelerating evidences suggest that necroptosis plays an important role in PDT-induced cell death in tumor cells (Miki et al., 2015; Sun et al., 2015), yet it is unknown whether necroptosis is involved in PDT-caused endothelial cell injury. In this study, we will analyze PDT-triggered cell death using a choroid-retinal endothelial cell line; and since rani is the only anti-VEGF antibody that received marketing authorisation for the treatment of ophthalmic diseases, we will compare the influences of combined treatment with rani at different time points.

## Materials and Methods

### Cell Culture

The rhesus macaque choroid-retinal endothelial (RF/6A) cell line was obtained from Chinese Academy of Sciences (Shanghai, China), and authenticated by STR profiling. RF/6A cells were grown in Dulbecco’s modified Eagle’s medium (DMEM) supplemented with 10% fetal bovine serum (FBS), 100 U/mL penicillin, and 100 U/mL streptomycin (all from Gibco, Rockville, MD, United States). The culture was incubated in 37°C humidified atmosphere with 5% CO_2_. RF/6A cells were used within 20 passages of the original vial, and MycoAlert Mycoplasma Detection Kit (Lonza, Basel, Switzerland) was used routinely to check mycoplasma contamination.

### PDT Treatment and Drug Administration

RF/6A cells were planted in a 96-well plate and kept overnight, and cell culture medium was changed into DMEM supplemented with 2% FBS just before drug administration. Verteporfin (Novartis, Basel, Switzerland), a photosensitizer for PDT, was added into the culture medium for 10 min. After washed with PBS twice, cells were incubated with fresh DMEM containing 2% FBS, and exposed to 689 nm laser (Opal photoactivator; Lumenis, Yokneam, Israel) at an intensity of 50 J/cm^2^ for 83 s to produce PDT effect. Various detections were performed at 12 h after PDT.

Rani (Novartis) was added into the culture medium at 6, 48 h before PDT or 6 h after PDT, and the other drugs right after PDT: Nec-1s (Selleckchem, Houston, TX, United States; 10 μM) is a potent and selective RIP1 inhibitor (Takahashi et al., 2012) and GSK’872 (Selleckchem; 1 μM) a specific RIP3 inhibitor (Mandal et al., 2014); necrosulfonamide (NSA; Selleckchem; 1 μM) was applied to inhibit the function of p-MLKL (Wang et al., 2014); MCC950 (Selleckchem; 1 μM) is a selective NLRP3 inhibitor (Zhang et al., 2017); Z-IETD-FMK (FMK; Selleckchem; 10 μM) was used to inhibit the activity of caspase 8 (cas8) (Luo et al., 2018); Kp7-6 (Calbiochem, San Diego, CA, United States; 1 mM) is a specific Fas/FasL antagonist (Meyer Zu Horste et al., 2018); R-7050 is a selective TNF-α receptor antagonist (Calbiochem; 10 μM) (Pajuelo et al., 2018); and batimastat (BB-94; Selleckchem; 10 μM) is a potent, broad spectrum matrix metalloprotease (MMP) inhibitor (Hogarth et al., 2019).

### Cell Viability Assay

Cell viability assays were performed by adding cell counting kit-8 (CCK-8; Dojindo, Kumamoto, Japan) directly into the culture medium. After incubation for 1 h at 37°C, the absorbance of the formazan dye was read by a spectrophotometer (Thermo Scientific, Waltham, MA, United States) at a wavelength of 450 nm. The absorbance data were normalized to Ctrl. Each experiment was repeated four times in three replicates.

### Flow Cytometry Analysis of Cell Apoptosis and Necrosis

Cell apoptosis and necrosis were tested by flow cytometry (Beckman Coulter, Brea, CA, United States) with Annexin-V-FITC/PI apoptosis detection kit (BD Biosciences, San Jose, CA, United States). In brief, RF/6A cells from each group were harvested by the end of cell treatment, and resuspended in binding buffer at a concentration of 1 × 10^6^ cells/ml. The cells were stained with Annexin-V-FITC and PI for 15 min in the dark at room temperature before flow cytometry performance. A minimum of 10,000 cells were analyzed in each sample with excitation/emission wavelengths at 494/525 nm and 536/617 nm.

### ELISA Assay

Cell culture medium supernatants from each group were collected by the end of treatment, and IL-1β, TNF-α, FasL and MMP-7 protein levels in supernatants were measured using monkey IL-1β, TNF-α, FasL and MMP-7 ELISA kits (all from LifeSpan BioSciences, Seattle, WA, United States) according to the manufacturer’s instructions. After adding stop solution to terminate reaction, the absorbance of substrate was read by a spectrophotometer at a wavelength of 450 nm immediately. Each experiment was performed four times in three replicates.

### Cellular Plasma Membrane Protein Extraction

Cellular plasma membrane proteins were extracted and purified using the Plasma Membrane Protein Extraction Kit (Abcam, Cambridge, United Kingdom). Cells were collected and homogenized in the Homogenize Buffer Mix. After centrifugation at 700 *g* for 10 min, the supernatants were transferred and centrifuged at 10,000 *g* for 30 min. The total membrane proteins pellet was re-suspended in 200 μl of the Upper Phase Solution and mixed with 200 μl of the Lower Phase Solution. After centrifugation at 1,000 *g* for 5 min, the upper phase was transferred to a new tube, and the lower phase was mixed with 100 μl of the Upper Phase Solution and centrifuged at 1,000 *g* for 5 min. The two upper phases were combined, mixed with 100 μl of the Lower Phase Solution and centrifuged at 1,000 *g* for 5 min. The upper phase was diluted with 5 volume of water and spun at top speed for 10 min, and the resulting pellet is the plasma membrane proteins.

### Immunoblotting Analyses

Cells from each group were harvested and lysed in RIPA buffer (50 mmol/L Tris–HCl, pH 8.0, 1% NP-40, 1% sodium deoxycholate, 0.1% SDS and 150 mmol/L sodium chloride) supplemented with protease inhibitors (Roche, Basel, Switzerland), dithiothreithol (1 mmol/L), EDTA (1 mmol/L) and phenylmethanesulfonyl fluoride (0.1 mmol/L). Samples of cell lyses or purified plasma membrane proteins (10–30 μg) were resolved in 8–12% SDS-PAGE gels and transferred onto PVDF membrane (Bio-Rad Laboratories, Hercules, CA, United States). The membrane were blocked before incubated overnight at 4°C with rabbit antisera against RIP1 (1:1000; Cell Signaling Technology, Danvers, MA, United States), RIP3 (1:1000; Abcam), phosphorylated MLKL at Ser358 (p-MLKL; 1:1000; Abcam), cleaved caspase 3 (c-cas3; 1:1000; Cell Signaling Technology), NOD-like receptor family pyrin domain containing 3 (NLRP3; 1:1000; Cell Signaling Technology), c-cas1 (1:1000; Cell Signaling Technology), c-cas8 (1:2000; Novus Biologicals, Centennial, CO, United States), cas8 (1:1000; Cell Signaling Technology), TNF-α (1:1000; Abcam), Fas ligand (FasL; 1:1000; Abcam), or mouse antibodies against β-actin (1:1000; Abcam) and Na^+^/K^+^ ATPase (1:1000; Cell Signaling Technology). Next, the membranes were washed and incubated with horseradish peroxidase-conjugated secondary anti-rabbit IgG or anti-mouse IgG (both 1:2000; Cell Signaling Technology) for 1 h at room temperature. Peroxidase activity was visualized with the ECL kit (Millipore, Burlington, MA, United States). Images were taken and analyzed by the Gel Documentation Systems (Bio-Rad Laboratories).

### RNA Extraction, RT-PCR and Real-Time PCR

Cells from each group were collected by the end of treatment, and total RNA was extracted with Trizol reagent (Invitrogen, Carlsbad, CA, United States) following the manufacturer’s instruction. RNA (2 μg) was reverse-transcribed using PrimeScript RT reagent Kit (Takara Bio, Shiga, Japan), and cDNA was amplified by quantitative real-time PCR with SYBR Premix Ex Taq Kit (Takara Bio) and data calculated using the DCt method (2^–*DDCt*^). The following primers were used: RIP3, 5′-TGGTGTCCATCGAGGAAC-3′ and 5′-GTCCCAGTTCACCTTCTC-3′; TNF-α, 5′-GTTCCTCAGCC TCTTCTCCT-3′ and 5′-ATCTGTCAGCTCCACGCCAT-3′; FasL, 5′-CAGAAGGCCTGGTCAAAGG-3′ and 5′-ACTCTCG GAGTTCTGCCAG-3′; GAPDH, 5′-CATCACTGCCACCC AGAA-3′ and 5′- ACGCCTGCTTCACTACCT-3′. Each experiment was repeated four times in three replicates.

### Cas8 Knockdown With Small Interfering RNA (siRNA)

The targeting sense sequence for cas8 knockdown in RF/6A cells is 5′-ATGCCTTGATGTTATTCCAGAGACT-3′ (Invitrogen). Stealth RNAi Negative Control Duplex (Medium GC, Invitrogen) was applied as a negative control. They were both transfected with Lipofectamine RNAi MAX (Invitrogen) following the manufacturer’s instruction. Cells were used for various experiments 2-day after transfection.

### Caspase Activity Assay

Cas3 and cas8 activitiy was measured using cas3 and cas8 colorimetric activity assay kits (BioVision, Inc., Milpitas, CA, United States). Briefly, cells from each group were collected, lysed and centrifuged. Caspase activities in the supernatants were determined by measuring the absorbance of the colorimetric substrate DEVD-pNA (cas3 activity) and IETD-pNA (cas8 activity) using a spectrophotometer at a wavelength of 405 nm before and after incubation at 37°C for 1 h. The protein concentration of each sample was assayed, and one unit of enzyme activity was defined as an absorbance change of 1 μg protein/h. Each experiment was performed four times in three replicates.

### MMP Activity Assay

By the end of treatment, cells from each group were harvested and subjected to 5 cycles of freeze-thaw. Cellular membrane pellets were collected after centrifugation at 20,000*g* for 15 min at 4°C, and used for TACE activity detection by SensoLyte 520 TACE (α-Secretase) Activity Assay Kit (AnaSpec, Fremont, CA, United States). Meanwhile, cell culture medium supernatants from each group were collected and MMP-7 activity in supernatants was measured with SensoLyte 520 MMP-7 Assay Kit (AnaSpec) following the manufacturer’s instruction. After adding stop solution to terminate reaction, the 5-FAM fluorescence intensity from each well was measured at Ex/Em = 490 nm/520 nm. The substrate control well fluorescence reading accounts for the background fluorescence, which was subtracted from the readings of the other wells. The resulting data were relative fluorescence units (RFU), and TACE or MMP-7 activity was expressed as RFU/μg protein. Each experiment was repeated four times in three replicates.

### Statistical Analysis

The data are presented as means ± SEM and were subjected to statistical analysis through one-way or two-way ANOVA, followed by Bonferroni *post hoc* analysis, with GraphPad Prism software (GraphPad Software, Inc., San Diego, CA, United States). The level of statistical significance was set at *P* < 0.05.

## Results

### Pre-compared to Post-treatment With Rani Significantly Aggravates PDT Injury in RF/6A Cells

**FIGURE 1:**
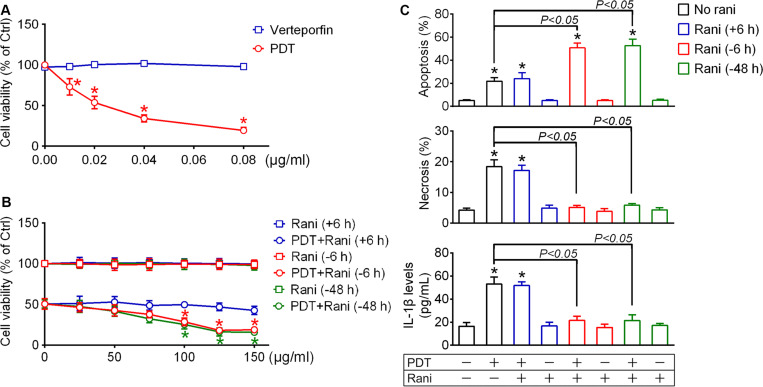
Pre-compared to post-treatment with ranibizumab (rani) aggravates photodynamic therapy (PDT) injury and alleviates inflammatory response in RF/6A cells. **(A)** For PDT treatment, RF/6A cells were treated with different doses of the photosensitizer verteporfin (0.01, 0.02, 0.04, and 0.08 μg/mL) for 10 min prior to irradiation with 689 nm laser (50 J/cm^2^) for 83 s. Cell viability was measured at 12 h using CCK-8 assay. **(B)** The anti-vascular endothelial cell growth factor (VEGF) antibody rani of various final concentrations (0, 25, 50, 75, 100, 125, and 150 μg/mL) were applied to PDT-exposed cells or normal cells at different time points [6, 48 h before PDT (-6, -48 h) or 6 h after PDT (+6 h)], and cell viability was also detected at 12 h after PDT. *n* = 4; *P < 0.05, compared with the control at 0 μg/mL in the same group. **(C)** Rani (125 μg/mL) was applied to PDT-exposed cells or normal cells at different time points (+6, -6, -48 h), and cell apoptosis and necrosis were determined by flow cytometry at 12 h after PDT while IL-1β protein levels in culture medium supernatants were measured via ELISA. *n* = 4; *P < 0.05, compared with the control.

For PDT treatment, RF/6A cells were exposed to a photosensitizer verteporfin at various final concentrations (0.01, 0.02, 0.04, and 0.08 μg/mL) for 10 min and irradiated with 689 nm laser (50 J/cm^2^) for 83 s. Cell viability was determined at 12 h using CCK-8 assay. Though verteporfin itself caused little harm to cells at any concentration, it led to remarkable decrease in cell viability after irradiation in a dose-dependent fashion, suggesting that severe cell injury is induced after PDT treatment (Figure 1A). The lethal median dose of verteporfin for PDT treatment was around 0.02 μg/mL, which was used in our following experiments.

Next, anti-VEGF rani was applied to PDT-exposed cells at various doses (25, 50, 75, 100, 125, and 150 μg/mL) and at different time points (6, 48 h before PDT or 6 h after PDT), and cell viability was detected as described above at 12 h after PDT. Post-treatment with rani had little impact on PDT injury, even at the highest dose. Pre-treatment with rani at 6 or 48 h had similar effects: they both aggravated PDT injury dose-dependently, and plateaus were reached at the concentration of 125 μg/mL (Figure 1B). It indicates that pre- compared to post-treatment with rani significantly aggravates PDT injury in RF/6A cells, and we chose the dose of 125 μg/mL in the subsequent experiments.

### Pre-compared to Post-treatment With Rani Prevents Necroptosis Yet Promotes Apoptosis in PDT-Exposed Cells

Cell apoptosis and necrosis were assayed by flow cytometry at 12 h after PDT. As shown in Figure 1C, the percentages of apoptotic and necrotic cells were both significantly increased after PDT in RF/6A cells, neither of which was affected by post-treatment with rani. However, PDT-induced necrosis was totally suppressed while apoptosis was further promoted by pre-treatment with rani at 6 or 48 h, with no appreciable difference between the two pre-treatment groups. That implicates that PDT-initiated necrosis is a controllable form of cell death, possibly necroptosis.

**FIGURE 2:**
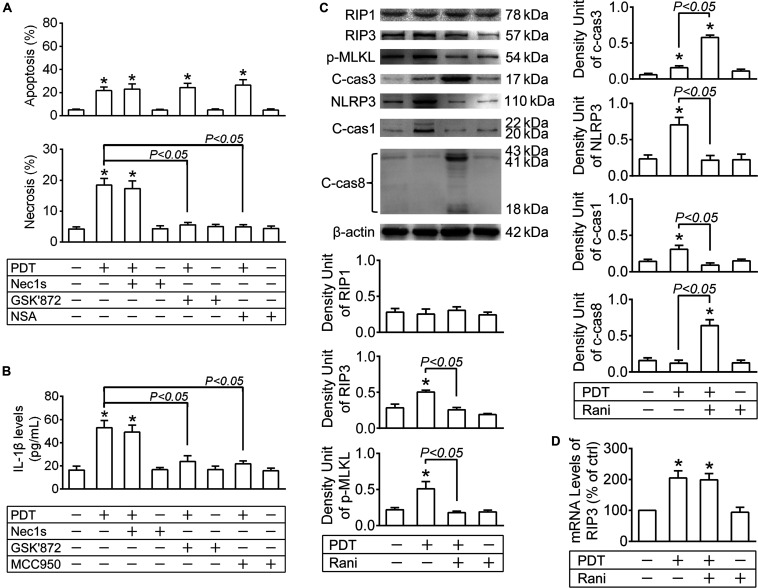
The impacts of PDT and pre-treatment of rani on apoptotic, necroptotic and NLRP3 inflammatory pathways in RF/6A cells. RF/6A cells were exposed to PDT, rani was applied to the cells at 6 h before PDT, and Nec1s (a specific receptor interacting protein kinase (RIP) 1 inhibitor), GSK’872 (a selective RIP3 inhibitor), necrosulfonamide (NSA, a specific p-MLKL inhibitor) and MCC950 (a selective NLRP3 inhibitor) were administrated right after PDT. Various detections were performed at 12 h after PDT. **(A)** Cell apoptosis and necrosis were determined by flow cytometry. **(B)** IL-1β protein levels in culture medium supernatants were measured via ELISA. **(C)** The protein levels of RIP1, RIP3, p-MLKL, c-cas3, NLRP3, c-cas1 and c-cas8 in RF/6A cells were detected by immnoblotting. **(D)** The mRNA levels of RIP3 were measured by real-time PCR analysis. *n* = 4; *P < 0.05, compared with the control.

To confirm that, the protein levels of the key players in necroptotic initiation (RIP1 and RIP3) and execution (p-MLKL) were detected by immunoblotting at 12 h after PDT. As seen from Figure 2C, RIP1 levels were barely affected in any group. RIP3 and p-MLKL levels were drastically elevated after PDT, which were both totally reversed by pre-treatment with rani at 6 h (Figure 2C) yet barely affected by rani post-treatment (Supplementary Figure S1). Meanwhile, the protein levels of the major apoptotic executioner c-cas3 were also measured, and they were remarkably increased after PDT, elevated even higher by rani pre-treatment (Figure 2C) but hardly affected by rani post-treatment (Supplementary Figure S1). Next, Nec1s (a specific RIP1 inhibitor), GSK’872 (a selective RIP3 inihibitor) or NSA (a specific p-MLKL inhibitor) was applied to RF/6A cells right after PDT, and cell apoptosis and necrosis were assayed at 12 h after PDT. As shown in Figure 2A, necrosis in PDT-exposed cells was completely suppressed by GSK’872 and NSA, yet barely affected by Nec1s. It confirms that necroptosis is involved in PDT injury in RF/6A cells, and it may be RIP3/p-MLKL-dependent but RIP1-independent.

The data above imply that PDT triggers necroptosis as well as apoptosis in RF/6A cells, while pre- compared to post-treatment with rani suppresses PDT-triggered necroptosis yet promotes apoptosis.

### Pre-compared to Post-treatment With Rani Prevents NLRP3/IL-1β Inflammatory Response in PDT-Exposed Cells

Necroptosis is accompanied with severe inflammation, partially because of released cellular contents acting on neighboring cells. Another reason is that aside from initiating necroptosis, RIP1 and RIP3 also play key roles in triggering NLRP3 inflammasome activation, including NLRP3 synthesis and cas1 activation, which eventually leads to IL-1β secretion (Moriwaki and Chan, 2016). To test the impact of PDT and combined treatment with rani on IL-1β secretion, IL-1β release was determined at 12 h after PDT by measuring IL-1β concentrations in the culture medium via Elisa. As seen from Figure 1C, IL-1β levels in the culture medium were elevated after PDT, which was completely suppressed by pre-treatment with rani at 6 or 48 h but not affected by post-treatment. No statistic difference was detected between the two pre-treatment groups.

The protein levels of NLRP3 and c-cas1 were detected by immunoblotting at 12 h after PDT. NLRP3 and c-cas1 levels were significantly elevated by PDT, which were both reversed by pre-treatment with rani at 6 h (Figure 2C) but hardly affected by rani post-treatment (Supplementary Figure S1), suggesting that pre-treatment with rani prevents PDT-induced NLRP3/IL-1β inflammatory response.

Next, Nec1s, GSK’872 and MCC950 (a NLRP3 inhibitor) were applied to RF/6A cells right after PDT, and IL-1β release was assayed at 12 h. IL-1β release from PDT-exposed cells was suppressed by GSK’872 and MCC950 but not Nec1s, indicating that it is probably RIP3/NLRP3-dependent but RIP1-independent (Figure 2B). Therefore, PDT-induced necoptosis and NLRP3/IL-1β inflammatory response RF/6A cells might be both mediated by RIP3.

### Cas8 Inhibition Abolishes Rani’s Effects of Aggravating PDT Injury and Alleviating NLRP3/IL-1β Inflammatory Response

Since the protein levels of RIP3 were increased by PDT, the mRNA levels of RIP3 were also tested at 12 h after PDT, and they were significantly elevated in PDT-exposed cells, implying that RIP3 generation may be upregulated after PDT (Figure 2D). Surprisingly, pre-treatment with rani at 6 h had little influence on the mRNA levels of RIP3 in PDT-treated cells (Figure 2D). Therefore, it is very likely that rani prevents PDT-caused RIP3 upregulation through accelerating degradation rather than interrupting production.

**FIGURE 3:**
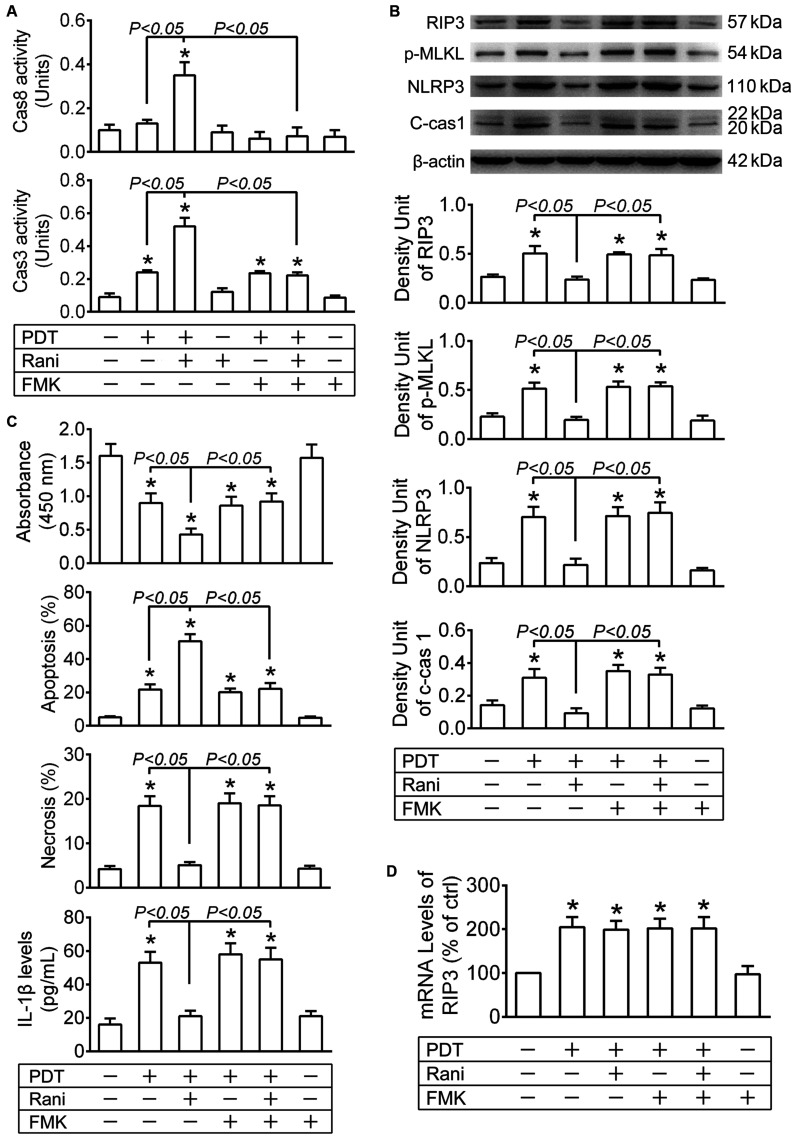
Cas8 inhibition reverses rani’s effects of preventing PDT-induced RIP3 upregulation and its downstream events. RF/6A cells were exposed to PDT, rani was applied to the cells at 6 h before PDT, and Z-IETD-FMK (FMK, a selective cas8 inhibitor) were administrated right after PDT. Various detections were performed at 12 h after PDT. **(A)** Cas3 and cas8 activitiy in RF/6A cells was measured using cas3 and cas8 colorimetric activity assay kits. **(B)** The protein levels of RIP3, p-MLKL, NLRP3 and c-cas1 in RF/6A cells were detected by immnoblotting. **(C)** Cell viability was tesed with CCK-8, cell apoptosis and necrosis were determined by flow cytometry, and IL-1β protein levels in cell culture medium were assayed using a monkey IL-1β ELISA kit. **(D)** The mRNA levels of RIP3 were measured by real-time PCR analysis. *n* = 4; *P < 0.05, compared with the control.

It has been suggested that RIP3 is a substrate of cas8, and it is rapidly degraded once cleaved by cas8 (Kang et al., 2013; Moriwaki and Chan, 2013). Firstly, the protein levels of activated cas8 (c-cas8) were determined by immunoblotting at 12 h after PDT. As shown in Figure 2C, c-cas8 levels were hardly affected after PDT, yet significantly increased by pre-treatment with rani at 6 h. Meanwhile, cas8 activity was assayed with a cas8 colorimetric activity assay kit, and similar results were observed (Figure 3A), indicating that pre-treatment with rani triggers cas8 activation in PDT-exposed cells. Next, cas8 activity was inhibited by FMK. Though FMK is a selective cas8 inhibitor, it has partial inhibition on cas3, especially at a high dose. The recommended concentration of FMK is 20 μM, and we chose a smaller dose (10 μM) and found that it provided a fine inhibition on cas8 activity and had no influence on cas3 activity (Figure 3A). FMK was applied right after PDT, the protein levels of RIP3 were measured at 12 h, and rani’s effect of preventing PDT-caused RIP3 upregulation was abolished by FMK (Figure 3B). FMK had no impact on the mRNA levels of RIP3 (Figure 3D). Those indicate that cas8 in PDT-exposed cells is highly activated by pre-treatment with rani, which might promote RIP3 degradation and prevent RIP3 upregulation.

Furthermore, rani’s effects of aggravating cell injury, switching necroptosis to apoptosis and preventing NLRP3/IL-1β inflammatory response in PDT-exposed cells were all reversed by FMK (Figures 3B,C), implying that those effects are likely mediated by activated cas8.

### Cas8 Knockdown Abolishes Rani’s Functions of Aggravating PDT Injury and Alleviating NLRP3/IL-1β Inflammatory Response

**FIGURE 4:**
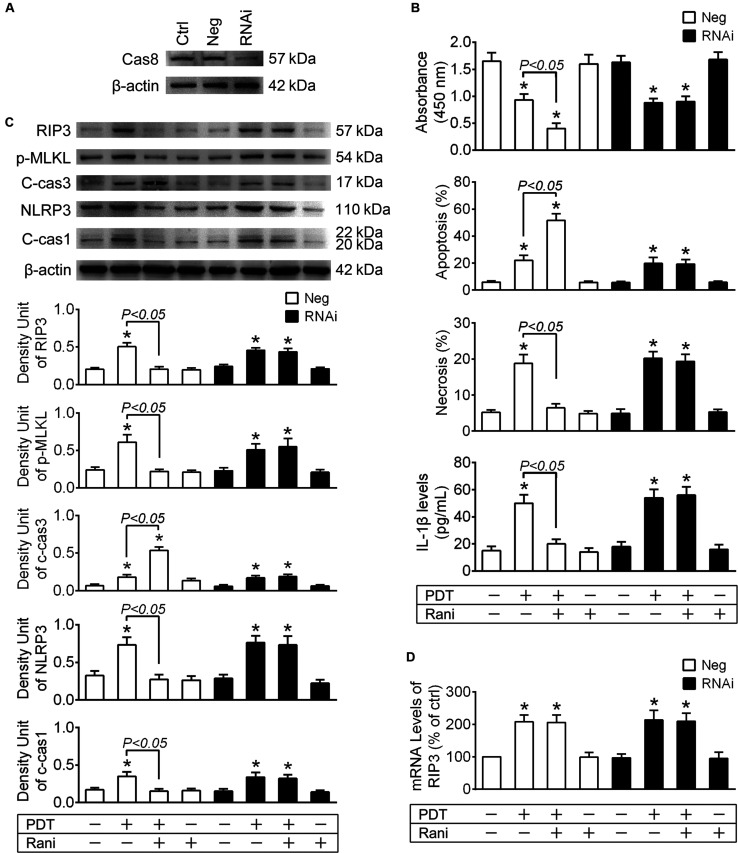
Cas8 knockdown abolishes rani’s function of suppressing RIP3 upregulation and its downstream events in PDT-exposed cells. **(A)** RF/6A cells were transfected with cas8 siRNA (RNAi group) or a negative control sequence (Neg group), and the expression of cas8 was assayed by immunoblotting at 2-day after transfection. **(B)** After cas8 knockdown for 2-day, RF/6A cells were exposed to PDT, and rani was applied to the cells at 6 h before PDT. Cell viability was measured with CCK-8 at 12 h after PDT, while cell apoptosis and necrosis were determined by flow cytometry, and IL-1β protein levels in cell culture medium were detected using a monkey IL-1β ELISA kit. **(C)** Meanwhile, the protein levels of RIP3, p-MLKL, c-cas3, NLRP3 and c-cas1 in RF/6A cells were detected by immnoblotting. **(D)** The mRNA levels of RIP3 were measured by real-time PCR analysis. *n* = 4; *P < 0.05, compared with the control.

To confirm the role of cas8 in rani’s function, RF/6A cells were transfected with cas8 siRNA or a negative control sequence, and the expression of cas8 was assayed by immunoblotting at 2-day after transfection. Compared with negative control sequence, Cas8 siRNA provided good knockdown of cas8 (Figure 4A) in RF/6A cells.

At 2-day after transfection, RF/6A cells were exposed to PDT, and similar results were observed that rani’s effects of increasing cell injury, switching necroptosis to apoptosis and suppressing NLRP3/IL-1β inflammatory response in PDT-exposed cells were all abolished after cas8 knockdown (Figures 4B,C). Cas8 knockdown itself had no further influence on the mRNA levels of RIP3 (Figure 4D).

The data from above confirm that cas8 may play a pivotal role in rani’s functions of aggravating cell injury and alleviating NLRP3/IL-1β inflammatory response in PDT-exposed cells.

### Pre-treatment With Rani Activates Cas8 in PDT-Exposed Cells Through Preventing MMP Activation and the Subsequent Cleavage of Death Ligands

**FIGURE 5:**
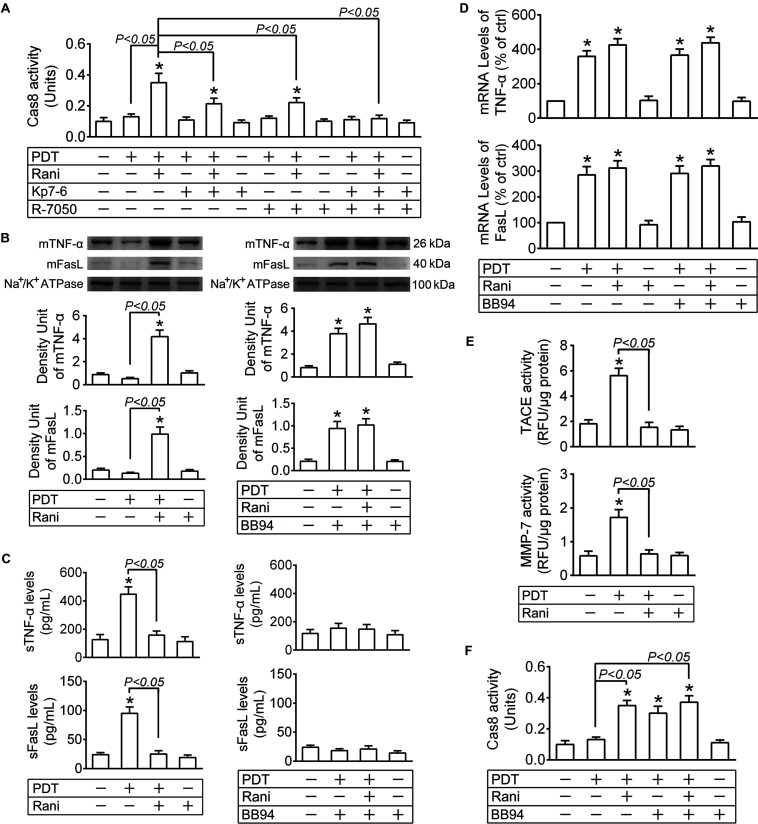
Pre-treatment with rani activates cas8 in PDT-exposed cells through preventing matrix metalloprotease (MMP)-mediated cleavage of death ligands. RF/6A cells were exposed to PDT, rani was applied to the cells at 6 h before PDT, and Kp7-6 (a specific Fas/FasL antagonist), R-7050 (a selective TNF-α receptor antagonist) and Batimastat (BB-94, a broad spectrum MMP inhibitor) were administrated right after PDT. Various detections were performed at 12 h after PDT. **(A,F)** Cas8 activity in the cells was measured with a cas8 colorimetric activity assay kit. **(B)** The plasma membrane proteins were extracted and purified, and the levels of membrane-bound ligands (mFasL and mTNF-α) were detected by immunoblotting. **(C)** The levels of soluble ligands (sFasL and sTNF-α) in cell culture medium supernatants were measured by ELISA. **(D)** The mRNA levels of FasL and TNF-α in RF/6A cells were tested by real time PCR. **(E)** MMP-7 activity in cell culture medium supernatants and TNF-α converting enzyme (TACE) activity in plasma membrane fraction were detected with SensoLyte 520 MMP-7 or TACE (α-Secretase) Activity Assay Kit. *n* = 4; *P < 0.05, compared with the control.

Cas8 occupies an essential position in the extrinsic apoptosis pathway triggered by a death ligand binding to its cell surface death receptor, such as Fas ligand (FasL)-Fas or tumor necrosis factor alpha (TNF-α)-TNF-α receptor (TNFR) (Tummers and Green, 2017). To explore whether cas8 activation triggered by rani is due to FasL-Fas or TNF-α-TNFR ligation, Kp7-6 or R-7050 was applied to RF/6A cells right after PDT, and cas8 activity was determined at 12 h. As shown in Figure 5A, rani-caused cas8 activation was partially prevented when FasL-Fas binding was interrupted by Kp7-6 or TNFR’s function was antagonized by R-7050, and it was completely suppressed after Kp7-6 and R-7050 were administrated together, implicating that FasL-Fas and TNF-α-TNFR might both be involved in rani-induced cas8 activation in PDT-exposed cells.

Upon translocation to the cell surface, membrane-bound ligands (mFasL and mTNF-α) undergo a proteolytic processing in their extracellular domains mediated by matrix metalloproteinases (MMPs), resulting in the release of soluble ligands (sFasL and sTNF-α) (Kayagaki et al., 1995; Mohan et al., 2002). Although mFasL and sFasL both bind to Fas, the former is far more capable of inducing apoptosis than the latter (Schneider et al., 1998), which is the same case with mTNF-α and sTNF-α (Ardestani et al., 2013). The plasma membrane proteins were extracted and purified at 12 h after PDT, and the levels of mFasL and mTNF-α were detected by immunoblotting; meanwhile, sFasL and sTNF-α levels in the cell culture medium supernatants were measured by ELISA. mFasL and mTNF-α levels were hardly affected by PDT yet both significantly elevated in PDT-exposed cells after pre-treatment with rani at 6 h (Figure 5B). However, sFasL and sTNF-α levels were dramatically increased after PDT, which were totally reversed by rani (Figure 5C). The mRNA levels of FasL and TNF-α in RF/6A cells were determined at the same time, and both of them were remarkably increased in PDT group, which were barely affected by rani (Figure 5D). It implies that PDT might result in FasL and TNF-α upregulation, which is not affected by pre-treatment with rani; nonetheless, those ligands are mostly in membrane-bound form in combined treatment group yet cleaved into soluble form in PDT group.

mFasL cleavage is mainly mediated by MMP-7 (Mitsiades et al., 2001) and sTNF-α release by TNF-α converting enzyme (TACE) (Mohan et al., 2002). The activities of both MMPs were tested at 12 h after PDT, and they were significantly elevated after PDT, which were entirely reversed by rani (Figure 5E). Next, BB94 was applied right after PDT to suppress MMP activities. PDT-caused increase in sFasL and sTNF-α levels was prevented by BB94 (Figure 5C), and meanwhile, mFasL and mTNF-α levels in the same group were remarkably elevated (Figure 5B), suggesting that the cleavage of mFasL and mTNF-α in PDT-exposed cells was hindered by BB94. Cas8 activity in PDT-treated cells was also remarkably elevated by BB94 (Figure 5F), confirming that membrane-bound death ligands are more capable of inducing apoptosis than the soluble ones. To exclude the impact of BB94 on ligand generation, the mRNA levels of FasL and TNF-α were also measured at 12 h after PDT, and BB94 had little influence on either of them (Figure 5D).

Those implicate that PDT leads to the upregulation of death ligands, which are yet unable to further activate cas8 due to their cleavage by activated MMPs. Pre-treatment with rani triggers cas8 activation in PDT-exposed cells via reducing MMP activities and the subsequent cleavage of death ligands.

## Discussion

Here, we showed for the first time that pre- compared to post-treatment with rani significantly aggravated PDT injury in RF/6A cells. PDT is a type of minimally invasive therapy based on the systemic administration of a photosensitizer and its local activation by light. The photosensitizer selectively accumulates in the abnormal blood vessels, and light irradiation induces generation of reactive oxygen species, resulting in local damage to the endothelium and blockage of the vessels (Kumar et al., 2019). Endothelial cell injury after light irradiation happens really quickly, which might explain why it was only affected by pre-treatment of rani whereas even post-treatment with rani at 6 h had little impact on it. However, our present work was performed only with cultured cells. Although RF/6A cells are a widely used endothelial cell line to model retinal and choroidal angiogenesis, its endothelial cell characteristics have been questioned by a recent study (Makin et al., 2018). More importantly, cultured cells could not entirely mimick the diseased endothelial cells in PCV, and the in vivo signaling mechanisms underlying PDT and rani treatment are more complicated and multifactorial. Therefore, our results need to be confirmed by in vivo experiments.

Our results in the present study demonstrated that apoptosis and necroptosis might be both involved in PDT injury in choroid-retinal endothelial cells. Cell apoptosis can be divided into two categories, both crucially dependent on the activation of caspases: intrinsic apoptosis is initiated by cas9 and extrinsic apoptosis by cas8. Those initiator caspases eventually activate the effector caspases, such as cas3, to carry out apoptosis (Slee et al., 2001; Keller et al., 2018). It has long been recognized that mitochondria play a central role in ROS-based PDT injury (Mroz et al., 2011). We also found here that cas8 was not activated after PDT, indicating that PDT-triggered apoptosis may be mainly mediated through the intrinsic mitochondrial pathway. Necroptosis initiation requires a cytosolic complex named the necrosome, and RIP1 and RIP3 are both known as components of necrosome; however, it has been suggested recently that RIP3 is indispensable for necroptosis, whereas RIP1 is not consistently required for the signal transduction (Liu et al., 2019). Similar results were observed in our present work, showing that the expression of RIP3 yet not RIP1 was selectively elevated after PDT, and PDT-induced necroptosis could be blocked by a RIP3 inhibitor but not a RIP1 inhibitor.

Aside from initiating necroptosis, RIP1 and RIP3 can also trigger activation of the NLRP3 inflammasome, thereby causing inflammatory response independently of necroptotic cell death by promoting the generation of proinflammatory cytokines such as IL-1β (Silke et al., 2015). Nevertheless, we demonstrated from above that PDT-caused IL-1β release could be prevented by RIP3 inhibition but not RIP1 inhibition. Those implicate that necroptosis and NLRP3 inflammasome activation after PDT might be both the downstream events of RIP3. Therefore, RIP3 is likely a potential target to alleviate PDT-triggered necroptosis as well as inflammatory response in choroid-retinal endothelial cells. That was confirmed by our results from above, that PDT-triggered necroptosis and NLRP3/IL-1β inflammatory response in RF/6A cells were both successfully suppressed by a RIP3 inhibitor. Nonetheless, cell apoptosis in PDT-exposed cells was barely affected by RIP3 at the same time, which makes the total cell death remarkably reduced. This indicates that RIP3 inhibition might reduce PDT-initiated necroptosis and inflammatory response at the risk of diminishing PDT’s angio-occlusive efficiency.

Intriguingly, we found in our present work that though rani also suppressed PDT-induced necroptosis, it could not rescue RF/6A cells from PDT injury. As a matter of fact, pre-treatment with rani even aggravated PDT injury, probably because cell apoptosis was significantly increased due to the activation of death receptor-mediated extrinsic apoptotic pathway. It has been reported that blocking the function of VEGF enhances the susceptibility of endothelial cells to various kinds of injury (Domigan et al., 2015; Watson et al., 2016). Since rani itself had no effect on cas8 activity, it is likely that rani pre-treatment further increases apoptosis through VEGF-deprivation. Despite marked differences between apoptosis and necroptosis, they are highly interconnected, especially at the initiation stage (Morris et al., 2017; Songane et al., 2018). Emerging evidence has suggested that cas8 stands at the crossing of apoptosis and necroptosis, and activated cas8 initiates apoptosis while it suppresses necroptosis via causing the direct cleavage of RIP3 and interrupting necrosome formation (Mocarski et al., 2011; Kang et al., 2013). We demonstrated here that cas8 was activated in PDT-exposed cells by pre-treatment with rani, and cas8 inhibition or knockdown completely abolished rani’s function of promoting apoptosis and total cell death, reversing RIP3 upregulation and suppressing necroptosis and NLRP3/IL-1β inflammatory response. It implies that cas8 may play a pivotal role in all those functions of pre-treatment with rani. Therefore, cas8 might be a better target than RIP3 at alleviating PDT-induced necroptosis as well as inflammatory response in choroid-retinal endothelial cells, for that cas8 acts upstream of RIP3 and cas8 activation could meanwhile enhance PDT’s angio-occlusive efficiency.

**FIGURE 6:**
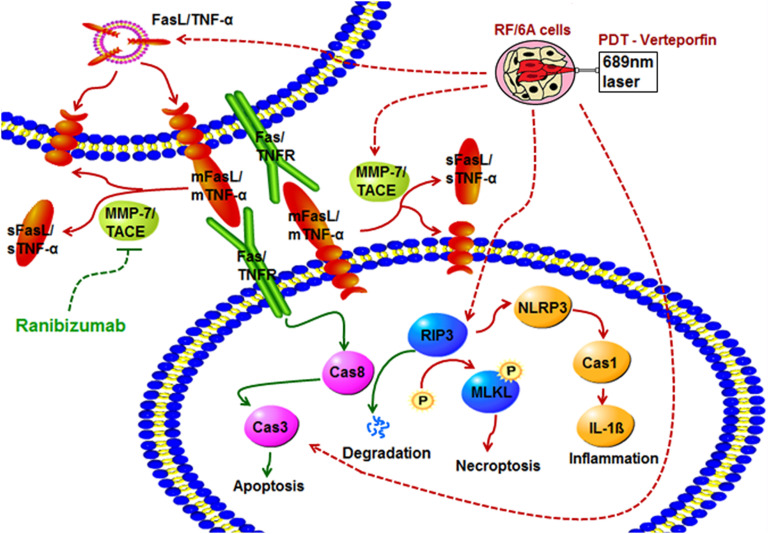
The effects of pre-treatment with rani on PDT injury in RF/6A cells and possible signaling pathways involved. PDT activates apoptosis, necroptosis and NLRP3 inflammasome in RF/6A cells. Pre-treatment with ranibizumab promotes PDT-caused apoptosis via triggering caspase 8-mediated extrinsic apoptosis, and caspase 8 might also play a pivotal role in the ranibizumab’s function of suppressing PDT-induced necroptosis and NLRP3 inflammasome activation.

In conclusion, we found here that pre- compared to post-treatment with rani significantly aggravates PDT injury in RF/6A cells. PDT activates apoptosis, necroptosis and NLRP3 inflammasome in RF/6A cells. Pre-treatment with rani promotes PDT-caused apoptosis via triggering caspase 8-mediated extrinsic apoptosis, and caspase 8 might also play a pivotal role in the rani’s function of suppressing PDT-induced necroptosis and NLRP3/IL-1β inflammatory response (Figure 6). Our results implicate that pre-treatment with rani may also have extra effects on PDT injury, which is to enhance the angio-occlusive efficiency of PDT and alleviate endothelial inflammatory response. This present work shed some new light into the mechanism underlying combined treatment with PDT and anti-VEGF, which might contribute to establish the optimal treatment protocol for PCV.

## Data Availability Statement

The raw data supporting the conclusions of this article will be made available by the authors, without undue reservation.

## Author Contributions

YL performed most part of the experiments. MZ, RG, and XW performed part of the experiments. MZ analyzed the data and drafted the article. LL and GX designed the study and revised the article. All authors contributed to the article and approved the submitted version.

## Conflict of Interest

The authors declare that the research was conducted in the absence of any commercial or financial relationships that could be construed as a potential conflict of interest.
