# Supplementary figures and images for: Pre-treatment With Ranibizumab Aggravates PDT Injury and Alleviates Inflammatory Response in Choroid-Retinal Endothelial Cells
Source: Front Cell Dev Biol. 2020 Jul 9;8:608. doi: 10.3389/fcell.2020.00608 (PMC7363772; doi:10.3389/fcell.2020.00608)

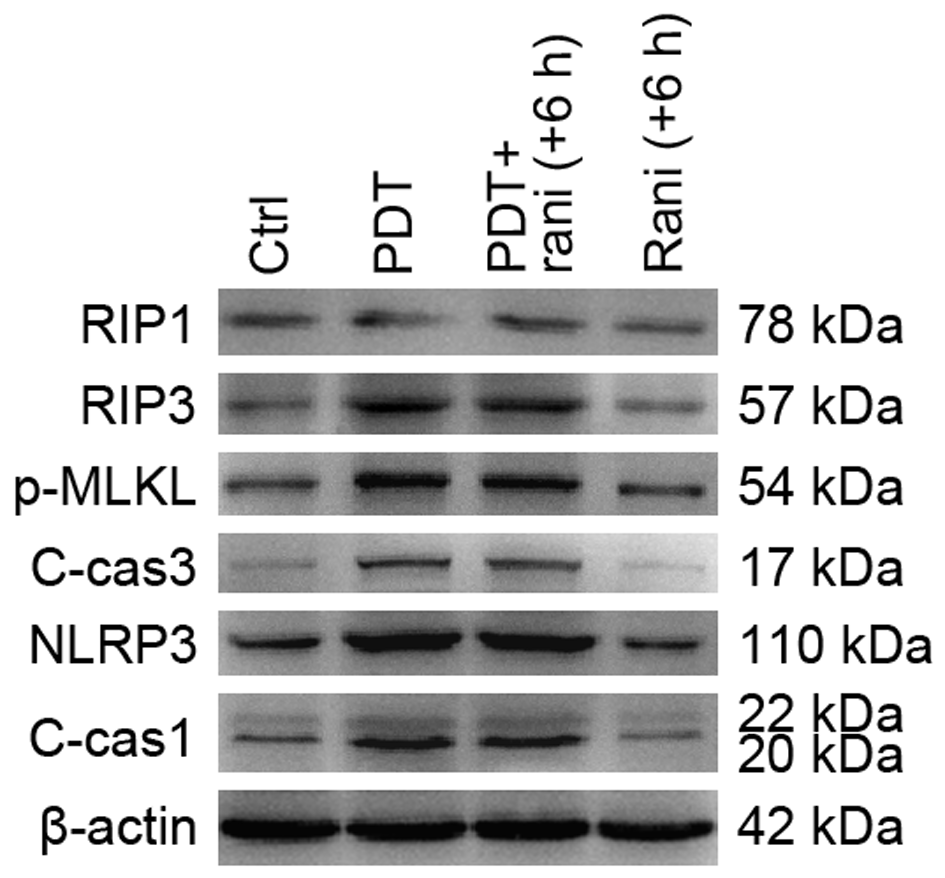

Supplement: Supplementary file 1 [file Figure_1.TIF]
